# Association between CHADS_2_ Score and the Preventive Effect of Statin Therapy on New-Onset Atrial Fibrillation in Patients with Acute Myocardial Infarction

**DOI:** 10.1371/journal.pone.0074709

**Published:** 2013-08-26

**Authors:** Shao-Sung Huang, Wan-Leong Chan, Hsin-Bang Leu, Po-Hsun Huang, Jaw-Wen Chen, Shing-Jong Lin

**Affiliations:** 1 Division of Cardiology, Department of Internal Medicine, Taipei Veterans General Hospital, Taipei, Taiwan; 2 Healthcare and Management Center, Taipei Veterans General Hospital, Taipei, Taiwan; 3 Department of Medical Research and Education, Taipei Veterans General Hospital, Taipei, Taiwan; 4 Institute of Clinical Medicine, National Yang-Ming University, Taipei, Taiwan; 5 Institute and Department of Pharmacology, National Yang-Ming University, Taipei, Taiwan; Universidad Peruana de Ciencias Aplicadas (UPC), Peru

## Abstract

**Objectives:**

New-onset atrial fibrillation (AF) commonly occurs in patients with acute myocardial infarction (AMI). Data regarding the value of the CHADS_2_ score in patients hospitalized for AMI is limited. This study aimed to determine whether the CHADS_2_ score is associated with new-onset AF and if it can help identify the patients who will benefit most from statin use for the prevention of arrhythmia after AMI.

**Methods:**

A total of 724 consecutive AMI patients were enrolled in this study. The patients were divided into 3 groups according to their CHADS_2_ scores: group 1, score 0; group 2, score 1–2; and group 3, score 3–6. The study endpoint was an episode of new-onset AF that lasted more than 30 seconds during hospitalization at the coronary care unit.

**Results:**

Seventy-eight (10.8%) patients developed new-onset AF, and 273 (37.7%) were on a statin upon admission. The incidence of new-onset AF increased significantly from 5.8% in group 1 to 11.3% in group 2 and 14.3% in group 3 (χ^2^ for linear trend, *P* = 0.017). Statin use (odds ratio [OR], 0.22; 95% CI, 0.06–0.85) and CHADS_2_ score (OR, 1.53; 95% CI, 1.02–2.28) were independent predictors of new-onset AF in AMI patients. Patients with CHADS_2_ score ≤2 had significantly reduced C-reactive protein level and lower risk of developing new-onset AF if they were taking statins (*P* < 0.05). Multivariate logistic regression analysis demonstrated the benefit of statin use for preventing new-onset AF in patients with CHADS_2_ scores ≤2 (OR, 0.34; 95% CI, 0.14–0.81).

**Conclusions:**

The CHADS_2_ score is a convenient scoring system for predicting the incidence of new-onset AF and may help in identifying the patients who will benefit most from statin use for the prevention of arrhythmia after AMI.

## Introduction

Preexisting or new-onset atrial fibrillation (AF) commonly occurs in patients with acute coronary syndrome (ACS) [1,2] and is associated with complications. Using data from patients with ACS, who were enrolled in the Global Registry of Acute Coronary Events, Mehta et al. found that preexisting and new-onset AF are associated with increased hospital morbidity and mortality as compared to ACS patients without any AF. However, only new-onset AF is an independent predictor of in-hospital adverse events in patients with ACS [3]. Moreover, AF is associated with a greater 30-day mortality (29.3% vs. 19.1%) and 1-year mortality (48.3% vs. 32.7%) in patients with acute myocardial infarction (AMI) [2]. AF is more commonly associated with AMI in older patients and in those with higher Killip class or left ventricular dysfunction [4]. Accumulating evidence indicates that apart from triggers, AF development and perpetuation depends on the electrical and structural remodeling of the atria [5]. Thus, many studies on pharmacological therapies have shifted to non-channel blocking drugs with pleiotropic properties that have the potential to alter the underlying atrial substrate without concomitant pro-arrhythmic effects [6,7].

3-Hydroxy-3-methylglutaryl coenzyme A reductase inhibitors (i.e., statins) are highly effective and widely used lipid-lowering agents. The beneficial effects of aggressive statin therapy in ACS as well as analyses of indexes of inflammation, oxidation, and thrombosis support the existence of relevant pleiotropic effects [8,9]. Although observational studies support the protective role of statins against AF in ACS patients [10], data recommending the use of statins to prevent AF are insufficient [11]. The CHADS_2_ score (i.e., congestive heart failure, hypertension, age >75 years, diabetes, and previous stroke/transient ischemic attack) is used for embolic risk stratification and guidance in the management of patients with AF [12]. Recent studies demonstrate that a higher CHADS_2_ score is associated with a risk of recurrence after catheter ablation of AF [13,14]. However, no published studies have investigated the role of the CHADS_2_ score in the prediction of new-onset AF in patients presenting with AMI. Therefore, this study aimed to determine whether the CHADS_2_ score is associated with new-onset AF and if it can help identify the AMI patients who will benefit most from statin use for the prevention of arrhythmia.

## Methods

### Study Population

This was a retrospective study of consecutive patients with AMI admitted to a coronary care unit (CCU) between May 2002 and December 2005. AMI was defined as detection of elevated troponin I level ≥0.1 ng/mL, accompanied by either typical chest pain for >30 min and/or electrocardiographic changes (including ischemic ST-segment depression, ST-segment elevation, or pathologic Q waves). A transthoracic echocardiogram was recorded in each patient. Before enrollment, each patient’s chart was reviewed in detail to gather data on medications, coronary risk factors, previous cardiovascular events, and other systemic diseases. Hypertension was defined as systolic blood pressure ≥140 mmHg, diastolic blood pressure ≥90 mmHg, or antihypertensive treatment. Diabetes mellitus was defined as fasting plasma glucose levels ≥126 mg/dL or the use of hypoglycemic agents. Serum creatinine levels >2 mg/dL was classified as renal insufficiency. Killip class I was defined as absence of heart failure, class II as presence of rales and/or jugular venous distension, class III as presence of pulmonary edema and class IV as cardiogenic shock. Patients with hyperthyroidism, rheumatic valvular disease, and chronic AF were excluded. To reduce patient selection bias, there was no age limit or other specific exclusion criteria. Among the 747 screened patients, 23 were excluded due to rheumatic valvular disease (n = 2) or chronic AF (n = 21). This study was approved by the research ethics committee of Taipei Veterans General Hospital. The informed consent requirement was waived by the Institutional Review Board because researchers only accessed retrospectively a de-identified database for analysis purposes.

### Risk score calculation

The CHADS_2_ score was calculated for each patient by assigning 1 point each for age >75 years, hypertension, diabetes mellitus, and previous heart failure and 2 points for a previous stroke or transient ischemic attack. The study patients were divided into 3 groups according to their CHADS_2_ scores: group 1, score 0; group 2, score 1–2; and group 3, score 3–6. These cutoff values were determined according to a previous study on the risk of stroke [15]. Group 1 (low risk), group 2 (intermediate risk), and group 3 (high risk) included 154, 416, and 154 patients, respectively.

### Intervention strategies

A diseased vessel was defined as a major epicardial artery with ≥50% stenosis. Revascularization was recommended for all patients with ≥70% diameter obstruction in any artery supplying a significant proportion of the myocardium. Percutaneous coronary intervention was recommended if there were 1 or 2 target lesions; meanwhile, coronary artery bypass grafting (CABG) was preferred in patients with 3-vessel or left main coronary artery disease (CAD).

### Clinical follow-up for endpoint

All patients were kept in the CCU for at least 5 days. During their stay at the CCU, all study subjects were followed up with continuous ECG monitoring for the occurrence of AF, which was defined as an irregular narrow complex rhythm with the absence of discrete P waves. The study endpoint was an episode of new-onset AF that lasted more than 30 seconds during hospitalization at the CCU.

### Statistical analysis

Data are expressed as mean ± standard deviation for numeric variables and numbers (percent) for categorical variables. Comparisons of continuous variables between groups were performed by Student’s t-test or one-way ANOVA test. Subgroup comparisons of categorical variables were assessed by the χ^2^ test or Fisher’s exact test. Univariate analysis was performed for analyzing the relationships between new-onset AF and clinical factors including statin use and CHADS_2_ score. Variables significantly associated with the presence of new-onset AF were entered into a multivariate regression model. Multivariate logistic regression analysis was performed to determine the independent predictors of new-onset AF. All data analyses were performed with SPSS software (version 17; SPSS, Chicago, IL, USA). The level of statistical significance was set at *P* < 0.05.

## Results

### Patient characteristics

**Figure 1:**
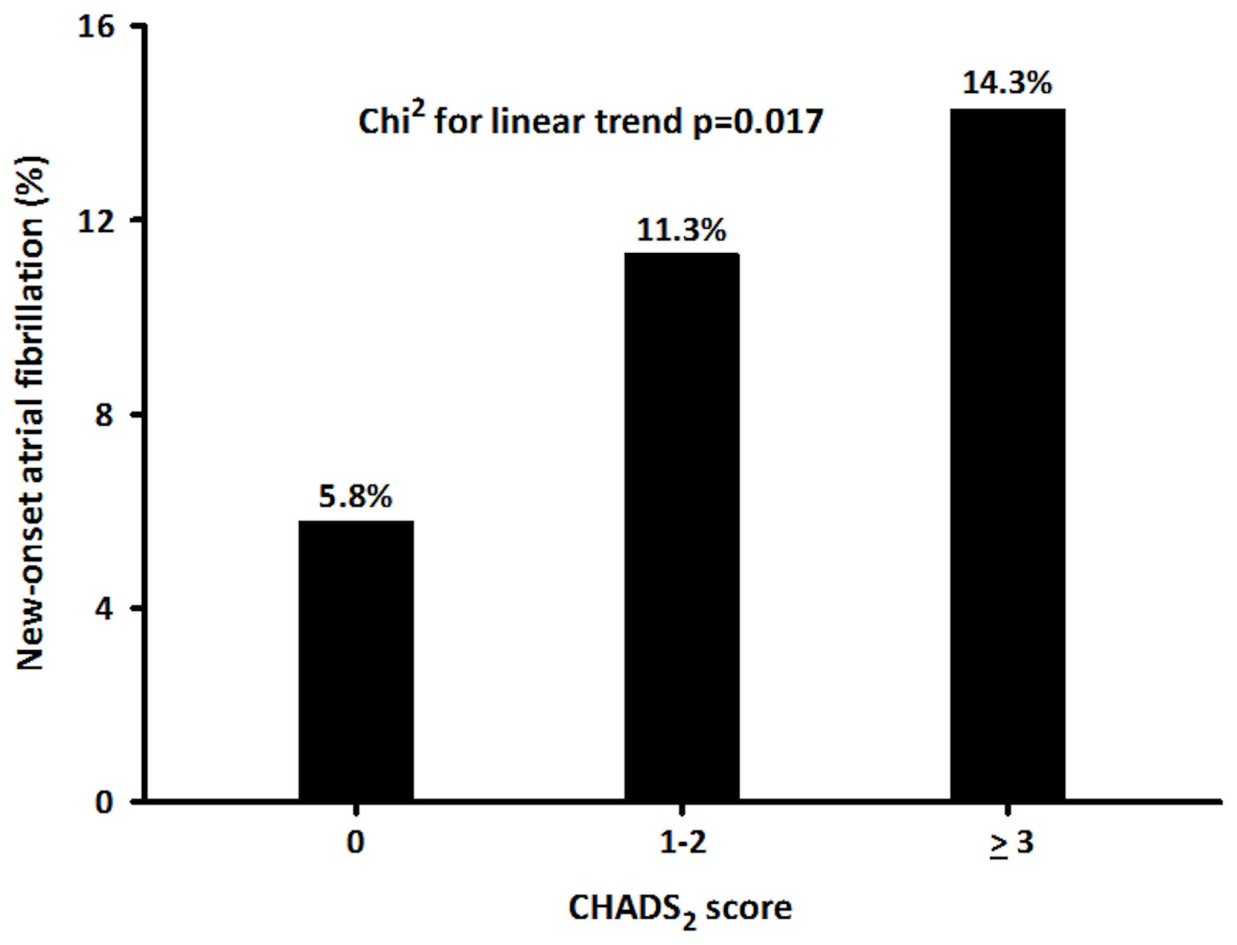
Incidence of new-onset AF in AMI patients according to CHADS_2_ score. AF, atrial fibrillation; AMI, acute myocardial infarction.

A total of 724 consecutive patients (582 men, 80%) were enrolled in this study. The mean age in our cohort was 67 ± 12 years. Seventy-eight (10.8%) developed new-onset AF, and 273 (37.7%) were on a statin at the time of admission. The baseline characteristics of all patients are shown in Table 1. Among the subjects, 64.1% had hypertension, 36.6% had diabetes mellitus, 18.4% had renal insufficiency, 12.2% had a previous stroke/transient ischemic attack, and 5.9% had previous heart failure. The CHADS_2_ scores of groups 1, 2, and 3 were 0, 1.46 ± 0.50, and 3.57 ± 0.70, respectively. Patients with high CHADS_2_ scores tended to be older and had increased left atrial (LA) diameter, lower left ventricular ejection fraction (LVEF), higher Killip classification, elevated C-reactive protein (CRP) level, and a higher frequency of underlying disease than patients with low CHADS_2_ scores. The incidence of new-onset AF increased significantly from 5.8% in group 1 to 11.3% in group 2 and 14.3% in group 3 (χ^2^ for linear trend, *P* = 0.017) (Figure 1).

**Table 1:** Baseline characteristics of patients according to CHADS_2_ scores.

	CHADS_2_ 0	CHADS_2_ 1–2	CHADS_2_ ≥3	
	(n = 154)	(n = 416)	(n = 154)	*P*
Age (years)	59.3 ± 11.1	67.1 ± 11.8	75.6 ± 7.7	<0.001
Male	140 (90.9)	336 (80.8)	106 (68.8)	<0.001
Hypertension	0 (0.0)	316 (76.0)	148 (96.1)	<0.001
Diabetes mellitus	0 (0.0)	159 (38.2)	106 (68.8)	<0.001
Renal insufficiency	10 (6.5)	76 (18.3)	47 (30.5)	<0.001
Previous PCI	8 (5.2)	45 (10.8)	23 (14.9)	0.019
Previous CABG	3 (1.9)	18 (4.3)	10 (6.5)	0.143
Previous MI	9 (5.8)	51 (12.3)	19 (12.3)	0.075
Previous stroke/TIA	0 (0.0)	4 (1.0)	84 (54.9)	<0.001
Previous heart failure	0 (0.0)	13 (3.1)	30 (19.5)	<0.001
Left atrial diameter (mm)	36.6 ± 4.9	39.7 ± 6.0	41.8 ± 7.5	<0.001
LVEF (%)	47.4 ± 13.7	43.5 ± 13.5	42.1 ± 13.5	0.011
Killip classification				<0.001
Killip = 1	116 (75.3)	237 (57.0)	55 (35.7)	
Killip > 1	38 (24.7)	179 (43.0)	99 (64.3)	
Medication use at index admission				
β-blocker	86 (55.8)	215 (51.7)	80 (51.9)	0.665
ACE inhibitor	104 (67.5)	255 (61.3)	73 (47.4)	0.001
Calcium channel blocker	6 (3.9)	120 (28.8)	70 (45.5)	<0.001
Statin	66 (42.9)	164 (39.4)	43 (27.9)	0.014
CRP (mg/dl)	0.83 ± 1.28	0.95 ± 1.51	1.33 ± 1.80	0.027
CHADS_2_ score	0	1.47 ± 0.50	3.57 ± 0.70	<0.001

Values are mean ± SD or number (%).

MI: myocardial infarction; PCI: percutaneous coronary intervention; CABG: coronary artery bypass grafting; TIA: transient ischemic attack; LVEF: left ventricular ejection fraction; ACE: angiotensin-converting enzyme; A II: angiotensin II; CRP: C-reactive protein

### Factors associated with new-onset AF

Patients with new-onset AF tended to be older, have increased LA diameter, have lower LVEF, have higher Killip classification, have elevated CRP level, and were more likely to have a higher CHADS_2_ score than patients without new-onset AF (all *P* < 0.05) (Table 2). In addition, new-onset AF occurred less frequently among statin users compared with nonusers (*P* < 0.001). Other demographic variables were similar between the groups.

**Table 2:** Baseline characteristics of patients with and without AF during hospitalization.

	With AF	Without AF	
	(n = 78)	(n = 646)	*p*
Age (years)	71.7 ± 10.0	66.7 ± 12.2	0.001
Male	66 (84.6)	516 (79.9)	0.319
Hypertension	54 (69.2)	410 (63.5)	0.316
Diabetes mellitus	31 (39.7)	234 (36.2)	0.542
Renal insufficiency	16 (20.5)	117 (18.1)	0.605
Previous PCI	13 (16.7)	63 (9.8)	0.060
Previous CABG	5 (6.4)	26 (4.0)	0.367
Previous MI	13 (16.7)	66 (10.2)	0.084
Previous stroke/TIA	14 (17.9)	74 (11.5)	0.098
Previous heart failure	7 (9.0)	36 (5.6)	0.211
Left atrial diameter (mm)	42.7 ± 8.3	39.3 ± 6.0	0.014
LVEF (%)	38.6 ± 15.2	44.6 ± 13.4	0.004
Killip classification			<0.001
Killip = 1	27 (34.6)	380 (58.9)	
Killip > 1	51 (65.4)	265 (41.1)	
Medication use at index admission			
β-blocker	33 (42.3)	348 (53.9)	0.053
ACE inhibitor	31 (39.7)	401 (62.1)	<0.001
Calcium channel blocker	17 (21.8)	179 (27.7)	0.267
Statin	12 (15.4)	261 (40.4)	<0.001
CRP (mg/dl)	1.52 ± 2.16	0.94 ± 1.42	0.043
CHADS_2_ score	2.05 ± 1.38	1.61 ± 1.26	0.004

Values are mean ± SD or number (%).

MI: myocardial infarction; PCI: percutaneous coronary intervention; CABG: coronary artery bypass grafting; TIA: transient ischemic attack; LVEF: left ventricular ejection fraction; ACE: angiotensin-converting enzyme; A II: angiotensin II; CRP: C-reactive protein

Table 3 lists the angiographic characteristics and interventional strategies of patients with and without new-onset AF. Coronary angiography was performed in 566 patients: 64 (82%) in the AF group and 502 (78%) in the non-AF group. Among patients with new-onset AF, insignificant CAD was found in 3 patients (4.7%), single-vessel disease in 5 (7.8%), and multi-vessel disease in 56 (87.5%). Patients with new-onset AF had significantly more CAD than those without new-onset AF (*P* < 0.001). Revascularization procedures were performed in 462 patients: 54 (69%) in the AF group and 408 (63%) in the non-AF group. The technique applied, either percutaneous or surgical, differed significantly between the two groups. Patients with new-onset AF underwent CABG more frequently than those without new-onset AF (48.1% vs. 11.0%, *P* < 0.001).

**Table 3:** Angiographic characteristics and revascularization strategies of patients with and without AF.

	With AF	Without AF	
	(n = 78)	(n = 646)	*P*
*Coronary angiography*	64	502	<0.001
Insignificant	3 (4.7)	26 (5.2)	
Single-vessel	5 (7.8)	159 (31.7)	
Multi-vessel	56 (87.5)	317 (63.1)	
*In-hospital revascularization*	54	408	<0.001
PCI	28 (51.9)	363 (89.0)	
CABG	26 (48.1)	45 (11.0)	

Values are mean ± SD or number (%).

PCI: percutaneous coronary intervention; CABG: coronary artery bypass grafting

### Independent predictors of new-onset AF

In order to investigate the independent predictors of new-onset AF in AMI patients, multivariate logistic regression analysis was performed with the following factors: CHADS_2_ score (i.e., congestive heart failure, hypertension, age >75 years, diabetes mellitus, and prior stroke or transient ischemic attack); serum levels of CRP; LA diameter; LVEF; Killip classification; extent of CAD; in-hospital CABG; and medications (i.e., ACE inhibitors, β-blockers, and statins). The use of statins (odds ratio [OR], 0.22; 95% CI, 0.06–0.85), LA diameter (OR, 1.08; 95% CI, 1.00–1.17), CHADS_2_ score (OR, 1.53; 95% CI, 1.02–2.28), and in-hospital CABG (OR, 4.42; 95% CI, 1.39–14.04) were independent predictors of new-onset AF in patients presenting with AMI (Table 4).

**Table 4:** Significant multivariate* predictors of new-onset atrial fibrillation.

	Odds ratio	95% CI	*P*
Statin use	0.223	0.059–0.849	0.028
Left atrial diameter (mm)	1.084	1.004–1.169	0.039
CHADS_2_ score	1.528	1.023–2.282	0.038
In-hospital CABG	4.422	1.393–14.04	0.012

CI: confidence interval; CABG: coronary artery bypass grafting

* Adjusted for CHADS_2_ score (i.e., congestive heart failure, hypertension, age > 75 years, diabetes mellitus, and prior stroke or transient ischemic attack); serum levels of C-reactive protein; left atrial diameter; left ventricular ejection fraction; Killip classification; in-hospital CABG; extent of coronary artery disease; and medications (i.e., ACE inhibitors, β-blockers, and statins).

### Relationship between CHADS_2_ score and effect of statins on new-onset AF

**Figure 2:**
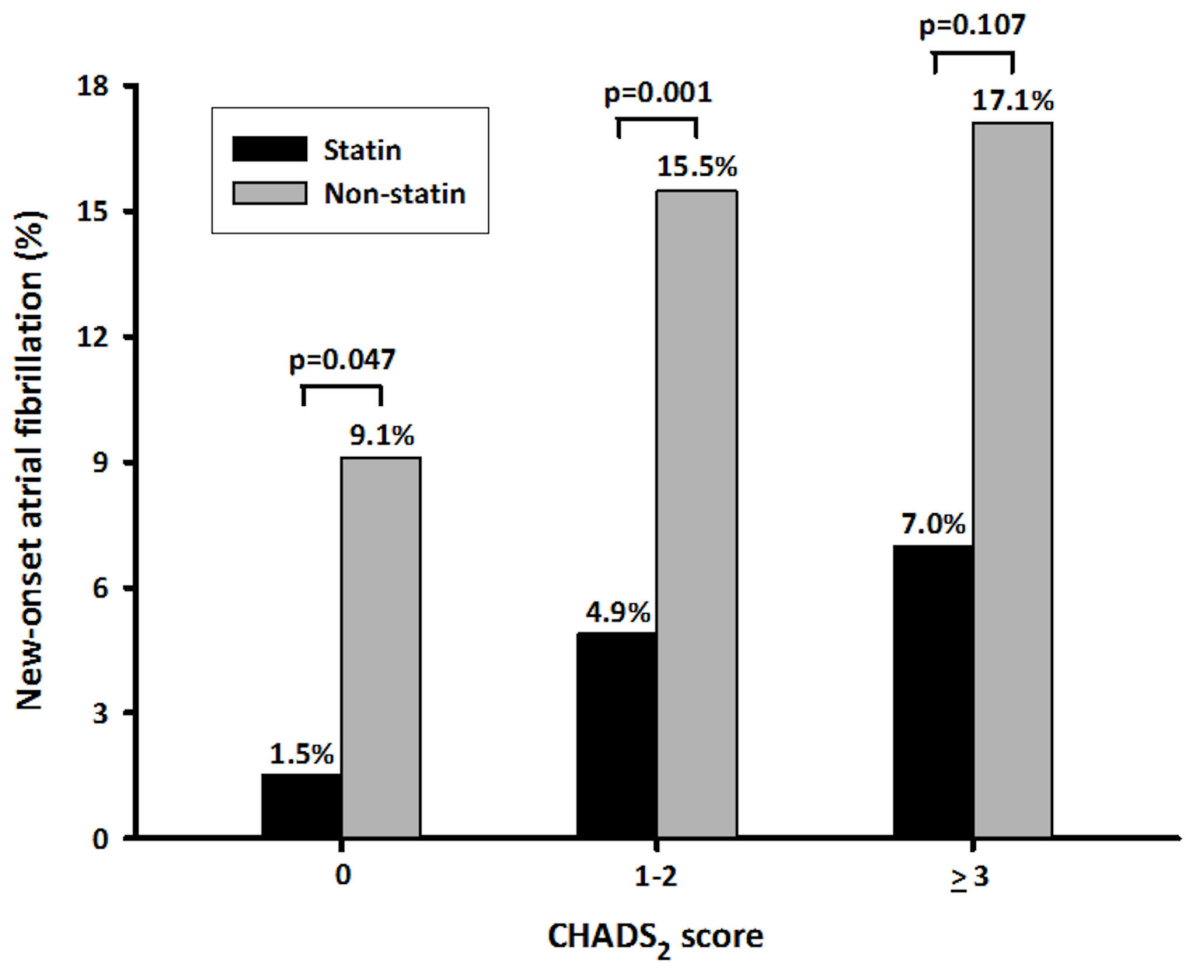
Relationship between CHADS_2_ score and the preventive effect of statin use on new-onset AF in patients with AMI.

**Figure 3:**
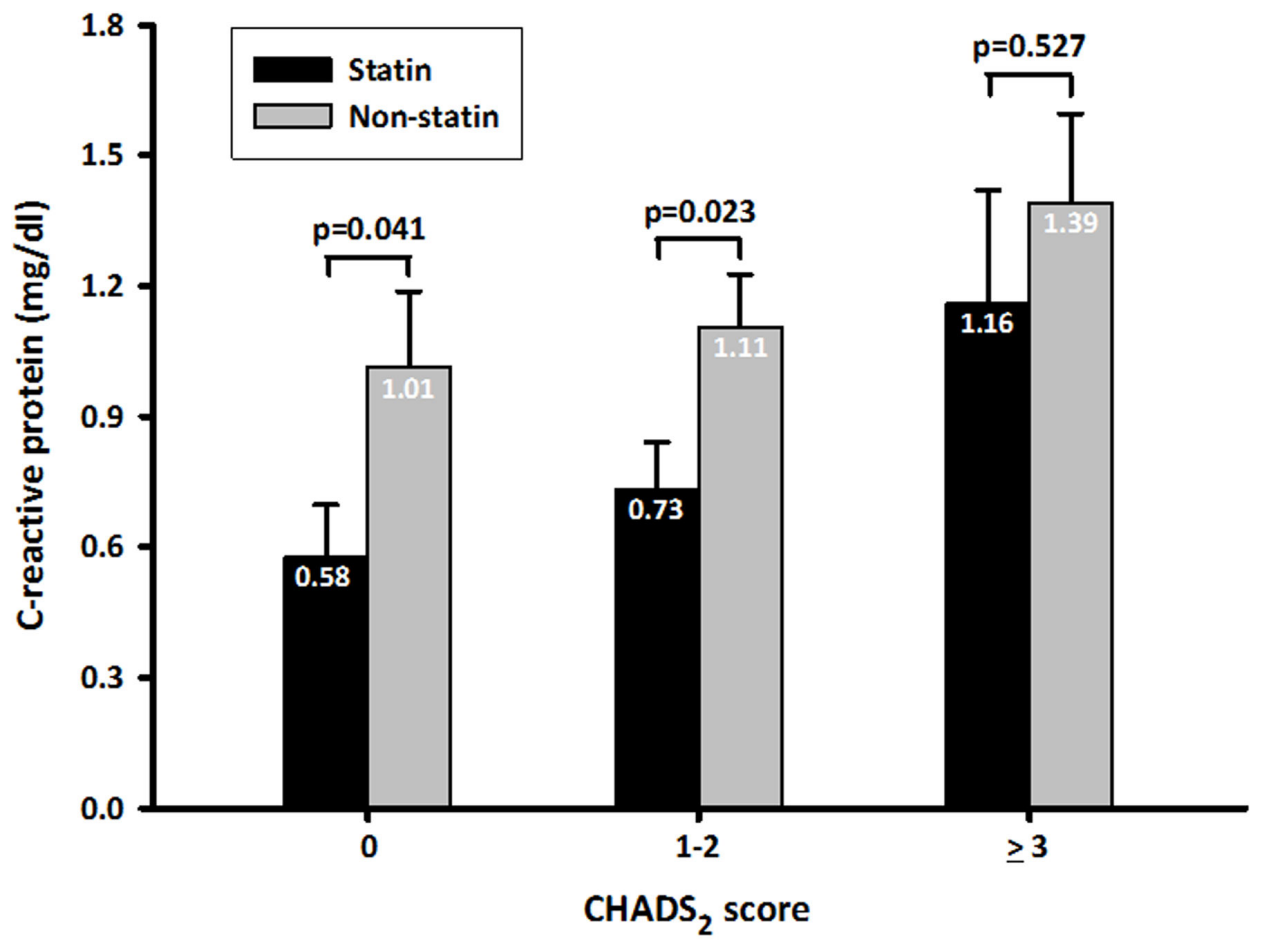
Relationship between CHADS_2_ score and the effect of statin therapy on CRP level in patients with AMI. CRP, C-reactive protein.

In the overall cohort, statin use was associated with a lower risk of developing new-onset AF. In patients with CHADS_2_ scores of 0, the incidence of new-onset AF was significantly lower in the statin group than that in the non-statin group (1.5% vs. 9.1%, *P* = 0.047) (Figure 2). Patients with CHADS_2_ scores of 1 or 2 also had a significantly lower risk of developing new-onset AF if they were taking statins (4.9% vs. 15.5%, *P* = 0.001). However, the benefit of statin use on the development of new-onset AF was not evident in patients with CHADS_2_ scores ≥3. Moreover, patients with CHADS_2_ scores ≤2 had significantly reduced CRP level if they were taking statins (*P* < 0.05) (Figure 3). The effect of statin therapy on CRP level was limited in patients with CHADS_2_ scores ≥3. Multivariate logistic regression analysis confirmed the benefit of statin use on new-onset AF in patients with CHADS_2_ scores ≤2 (OR, 0.34; 95% CI, 0.14–0.81).

## Discussion

The present results indicate that in a cohort of AMI patients, the incidence of new-onset AF was increased in patients with higher CHADS_2_ scores. Statin use was associated with a lower risk of developing new-onset AF. The effect of statin therapy on CRP level and new-onset AF was evident in patients with CHADS_2_ scores ≤2 but not in patients with high CHADS_2_ scores. These findings suggest that the CHADS_2_ score may help identify the AMI patients who will benefit most from statin use for the prevention of new-onset AF.

Some clinical observational and experimental studies suggest that the use of statins protects against AF. Additionally, a recent meta-analysis of 6 randomized controlled trials (3,557 patients) suggests that the use of statins is significantly associated with a decreased risk of the incidence or recurrence of AF in patients with sinus rhythm with a history of previous AF, undergoing cardiac surgery, or after ACS [16]. Our data are consistent with those in literature and demonstrate that statin treatment is associated with a lower incidence of new-onset AF in patients with AMI. There are various possible mechanisms of the antiarrhythmic effects of statins against AF. Accumulating evidence suggests that both inflammation and oxidative stress are involved in the development, recurrence, and persistence of AF [17,18]. These conditions are associated with enhanced myocardial tissue inflammation and atrial remodeling, which might serve as a substrate for the development of AF [19]. Moreover, elevated CRP levels are related to future AF development, AF persistence, and recurrence after cardioversion [20]. The capacity of statins to reduce inflammation, CRP levels, and oxidative stress is well-established [21–23]. This may explain the potential beneficial effect of statins against AF. Finally, statins may protect against AF in postoperative patients by modulating the autonomic nervous system against enhanced postoperative sympathetic activity [24], which increases susceptibility to AF. This could represent an alternative antiarrhythmic mechanism of statins against AF in such patients.

The CHADS_2_ score, which was initially developed for stroke risk stratification in AF patients, is a convenient scoring system for evaluating the complexity of comorbidities in patients. Previous study demonstrates that CHADS_2_ score is useful to predict CRP levels, LA thrombus formation, and prognosis in patients with nonrheumatic AF [25]. In the current study, we also showed that patients with high CHADS_2_ scores had elevated CRP levels and a higher frequency of underlying disease. The components of the CHADS_2_ score, including heart failure, hypertension, old age, and diabetes, are associated with an increased risk of the development of AF [11]. A recent study shows that high CHADS_2_ scores are associated with advanced atrial remodeling including structural (i.e., enlarged LA size) and electrophysiological (i.e., low LA voltage and prolonged activation time) changes, which result in recurrence after AF ablation [14]. In addition, the CHADS_2_ score is useful in risk estimation and stratification of new-onset AF [26]. Although there is limited knowledge of the value of the CHADS_2_ score in patients hospitalized for AMI, the CHADS_2_ score has several desirable characteristics for application in AMI patients because it is easily calculated at the bedside and includes clinical data routinely available in the CCU. The present results showed that the incidence of new-onset AF was significantly increased in AMI patients with higher CHADS_2_ scores: 5.8%, 11.3%, and 14.3% in groups 1, 2, and 3, respectively.

Furthermore, our results demonstrated that the CHADS_2_ score may help in identifying AMI patients who will benefit most from statin use for the prevention of new-onset AF. Among individuals presenting with AMI, those with CHADS_2_ scores ≤2 had a significantly lower risk of developing AF if they were taking statins (OR, 0.34; 95% CI, 0.14–0.81). However, patients with CHADS_2_ scores ≥3 did not exhibit similar benefits from statin therapy. Although the mechanism through which the CHADS_2_ score modifies the effect of statins on new-onset AF in AMI patients is unclear, high CHADS_2_ scores may be linked to a great burden of systemic inflammation that may attenuate the beneficial effect of statins against the development of AF. Indeed, our data showed that patients with CHADS_2_ scores ≤2 had significantly reduced CRP level if they were taking statins. However, the effect of statin therapy on CRP level was limited in patients with CHADS_2_ scores ≥3. Further prospective and large-scale trials are required to determine whether the aggressive treatment of the underlying disease decreases the incidence of new-onset AF in AMI patients with high CHADS_2_ scores.

### Study limitations

This study has some limitations that should be considered. First, the present study included a small population at a single center. The present findings should be confirmed in a large multicenter trial. Second, we only followed the patients during their stay at the CCU. New-onset AF occurring beyond this period will be missed. Third, although we were unable to determine how the CHADS_2_ score affects the relationship between statin therapy and the risk of developing AF, the mechanisms described above may partly explain the inverse correlation between the CHADS_2_ scores and effectiveness of statin use on new-onset AF in patients with AMI. Further studies are needed to clarify the exact interaction between statins and AF.

## Conclusions

New-onset AF is a common complication of AMI. Statin use is associated with a lower risk of developing AF in AMI patients. The CHADS_2_ score is a convenient scoring system for predicting the incidence of AF and may help in identifying the patients who will benefit most from statins for the prevention of arrhythmia after AMI.

## References

[1] Goldberg RJ, Yarzebski J, Lessard D, Wu J, Gore JM (2002) Recent trends in the incidence rates of and death rates from atrial fibrillation complicating initial acute myocardial infarction: A community-wide perspective. Am Heart J 143: 519–527. doi:10.1067/mhj.2002.120410. PubMed: 11868060. 11868060 10.1067/mhj.2002.120410

[2] Rathore SS, Berger AK, Weinfurt KP, Schulman KA, Oetgen WJ et al. (2000) Acute myocardial infarction complicated by atrial fibrillation in the elderly: prevalence and outcomes. Circulation 101: 969–974. doi:10.1161/01.CIR.101.9.969. PubMed: 10704162. 10704162 10.1161/01.cir.101.9.969

[3] Mehta RH, Dabbous OH, Granger CB, Kuznetsova P, Kline-Rogers EM et al. (2003) Comparison of outcomes of patients with acute coronary syndromes with and without atrial fibrillation. Am J Cardiol 92: 1031–1036. 14583352 10.1016/j.amjcard.2003.06.001

[4] Schmitt J, Duray G, Gersh BJ, Hohnloser SH (2009) Atrial fibrillation in acute myocardial infarction: a systematic review of the incidence, clinical features and prognostic implications. Eur Heart J 30: 1038–1045. doi:10.1093/eurheartj/ehn579. PubMed: 19109347. 19109347 10.1093/eurheartj/ehn579

[5] Shiroshita-Takeshita A, Brundel BJ, Nattel S (2005) Atrial fibrillation: basic mechanisms, remodeling and triggers. J Interv Card Electrophysiol 13: 181–193. doi:10.1007/s10840-005-2362-y. PubMed: 16177845. 16177845 10.1007/s10840-005-2362-y

[6] Page RL, Roden DM (2005) Drug therapy for atrial fibrillation: where do we go from here? Nat Rev Drug Discov 4: 899–910. doi:10.1038/nrd1876. PubMed: 16264433. 16264433 10.1038/nrd1876

[7] Murray KT, Mace LC, Yang Z (2007) Nonantiarrhythmic drug therapy for atrial fibrillation. Heart Rhythm 4: S88–S90. doi:10.1016/j.hrthm.2006.12.027. PubMed: 17336893. 17336893 10.1016/j.hrthm.2006.12.027

[8] Ray KK, Cannon CP, Ganz P (2006) Beyond lipid lowering: What have we learned about the benefits of statins from the acute coronary syndromes trials? Am J Cardiol 98: S18–S25. doi:10.1016/j.amjcard.2006.09.034. PubMed: 17126675. 10.1016/j.amjcard.2006.09.016 17126675

[9] Almuti K, Rimawi R, Spevack D, Ostfeld RJ (2006) Effects of statins beyond lipid lowering: potential for clinical benefits. Int J Cardiol 109: 7–15. doi:10.1016/j.ijcard.2005.05.056. PubMed: 16054715. 16054715 10.1016/j.ijcard.2005.05.056

[10] Ozaydin M, Turker Y, Erdogan D, Karabacak M, Dogan A et al. (2010) The association between previous statin use and development of atrial fibrillation in patients presenting with acute coronary syndrome. Int J Cardiol 141: 147–150. doi:10.1016/j.ijcard.2008.11.172. PubMed: 19106009. 19106009 10.1016/j.ijcard.2008.11.172

[11] Wann LS, Curtis AB, January CT, Ellenbogen KA, Lowe JE et al. (2011) 2011 ACCF/AHA/HRS focused update on the management of patients with atrial fibrillation (updating the 2006 guideline): A report of the American College of Cardiology Foundation/American Heart Association Task Force on Practice Guidelines. Circulation 123: 104–123. 21173346 10.1161/CIR.0b013e3181fa3cf4

[12] Gage BF, Waterman AD, Shannon W, Boechler M, Rich MW et al. (2001) Validation of clinical classification schemes for predicting stroke: results from the National Registry of Atrial Fibrillation. JAMA 285: 2864–2870. doi:10.1001/jama.285.22.2864. PubMed: 11401607. 11401607 10.1001/jama.285.22.2864

[13] Chao TF, Ambrose K, Tsao HM, Lin YJ, Chang SL et al. (2012) Relationship between the CHADS_2_ score and risk of very late recurrences after catheter ablation of paroxysmal atrial fibrillation. Heart Rhythm 9: 1185–1191. doi:10.1016/j.hrthm.2012.03.007. PubMed: 22406145. 22406145 10.1016/j.hrthm.2012.03.007

[14] Chao TF, Cheng CC, Lin WS, Tsao HM, Lin YJ et al. (2011) Associations among the CHADS_2_ score, atrial substrate properties, and outcome of catheter ablation in patients with paroxysmal atrial fibrillation. Heart Rhythm 8: 1155–1159. doi:10.1016/j.hrthm.2011.03.016. PubMed: 21402172. 21402172 10.1016/j.hrthm.2011.03.016

[15] Gage BF, van Walraven C, Pearce L, Hart RG, Koudstaal PJ et al. (2004) Selecting patients with atrial fibrillation for anticoagulation: Stroke risk stratification in patients taking aspirin. Circulation 110: 2287–2292. doi:10.1161/01.CIR.0000145172.55640.93. PubMed: 15477396. 15477396 10.1161/01.CIR.0000145172.55640.93

[16] Fauchier L, Pierre B, de Labriolle A, Grimard C, Zannad N et al. (2008) Antiarrhythmic effect of statin therapy and atrial fibrillation a meta-analysis of randomized controlled trials. J Am Coll Cardiol 51: 828–835. doi:10.1016/j.jacc.2007.09.063. PubMed: 18294568. 18294568 10.1016/j.jacc.2007.09.063

[17] Engelmann MDM, Svendsen JH (2005) Inflammation in the genesis and perpetuation of atrial fibrillation. Eur Heart J 26: 2083–2092. doi:10.1093/eurheartj/ehi350. PubMed: 15975993. 15975993 10.1093/eurheartj/ehi350

[18] Korantzopoulos P, Kolettis TM, Galaris D, Goudevenos JA (2007) The role of oxidative stress in the pathogenesis and perpetuation of atrial fibrillation. Int J Cardiol 115: 135–143. doi:10.1016/j.ijcard.2006.04.026. PubMed: 16764958. 16764958 10.1016/j.ijcard.2006.04.026

[19] Korantzopoulos P, Kolettis T, Siogas K, Goudevenos J (2003) Atrial fibrillation and electrical remodeling: the potential role of inflammation and oxidative stress. Med Sci Monit 9: RA225–RA229. PubMed: 12960937. 12960937

[20] Liu T, Li G, Li L, Korantzopoulos P (2007) Association between C-reactive protein and recurrence of atrial fibrillation after successful electrical cardioversion: A meta-analysis. J Am Coll Cardiol 49: 1642–1648. doi:10.1016/j.jacc.2006.12.042. PubMed: 17433956. 17433956 10.1016/j.jacc.2006.12.042

[21] Strandberg TE, Vanhanen H, Tikkanen MJ (1999) Effect of statins on C-reactive protein in patients with coronary artery disease. Lancet 353: 118–119. doi:10.1016/S0140-6736(05)76154-7. PubMed: 10023901. 10023901 10.1016/S0140-6736(05)76154-7

[22] Plenge JK, Hernandez TL, Weil KM, Poirier P, Grunwald GK et al. (2002) Simvastatin lowers C-reactive protein within 14 days: An effect independent of low-density lipoprotein cholesterol reduction. Circulation 106: 1447–1452. doi:10.1161/01.CIR.0000029743.68247.31. PubMed: 12234946. 12234946 10.1161/01.cir.0000029743.68247.31

[23] Rosenson RS (2004) Statins in atherosclerosis: lipid-lowering agents with antioxidant capabilities. Atherosclerosis 173: 1–12. doi:10.1016/S0021-9150(03)00239-9. PubMed: 15177118. 15177118 10.1016/S0021-9150(03)00239-9

[24] Liu T, Li GP (2006) Statins may prevent postoperative atrial fibrillation through autonomic modulation. Am J Cardiol 97: 1266. doi:10.1016/j.amjcard.2005.11.016. PubMed: 16616039. 16616039 10.1016/j.amjcard.2005.11.016

[25] Maehama T, Okura H, Imai K, Yamada R, Obase K et al. (2010) Usefulness of CHADS_2_ score to predict C-reactive protein, left atrial blood stasis, and prognosis in patients with nonrheumatic atrial fibrillation. Am J Cardiol 106: 535–538. doi:10.1016/j.amjcard.2010.03.067. PubMed: 20691312. 20691312 10.1016/j.amjcard.2010.03.067

[26] Chao TF, Liu CJ, Chen SJ, Wang KL, Lin YJ et al. (2012) CHADS(2) score and risk of new-onset atrial fibrillation: A nationwide cohort study in Taiwan. Int J Cardiol [Epub ahead of print]. PubMed: 23280330. 10.1016/j.ijcard.2012.12.011 23280330

